# Supplementary material for: Coapplication of Magnesium Supplementation and Vibration Modulate Macrophage Polarization to Attenuate Sarcopenic Muscle Atrophy through PI3K/Akt/mTOR Signaling Pathway
Source: Int J Mol Sci. 2022 Oct 26;23(21):12944. doi: 10.3390/ijms232112944 (PMC9654727; doi:10.3390/ijms232112944)
Supplement: Supplementary file 1 [file ijms-23-12944-s001.zip › ijms-1682742-supplementary.pdf]

Supplementary Material

Figures and Legends

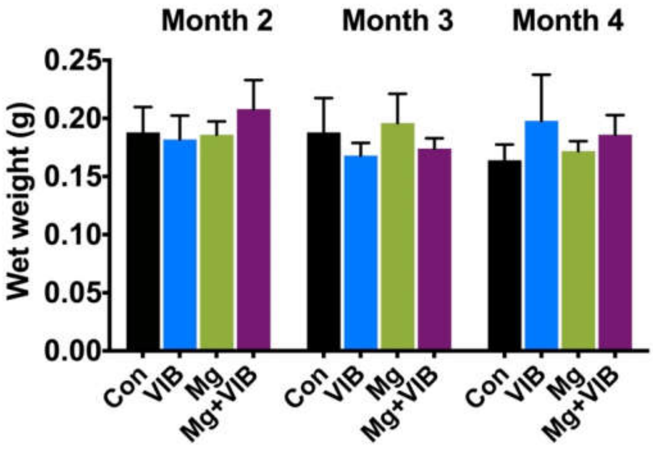

Figure S1. Wet weight of gastrocnemius among all treatment groups. Mg+VIB group showed higher wet weight than Con group at month 2, yet without significance (n=5).

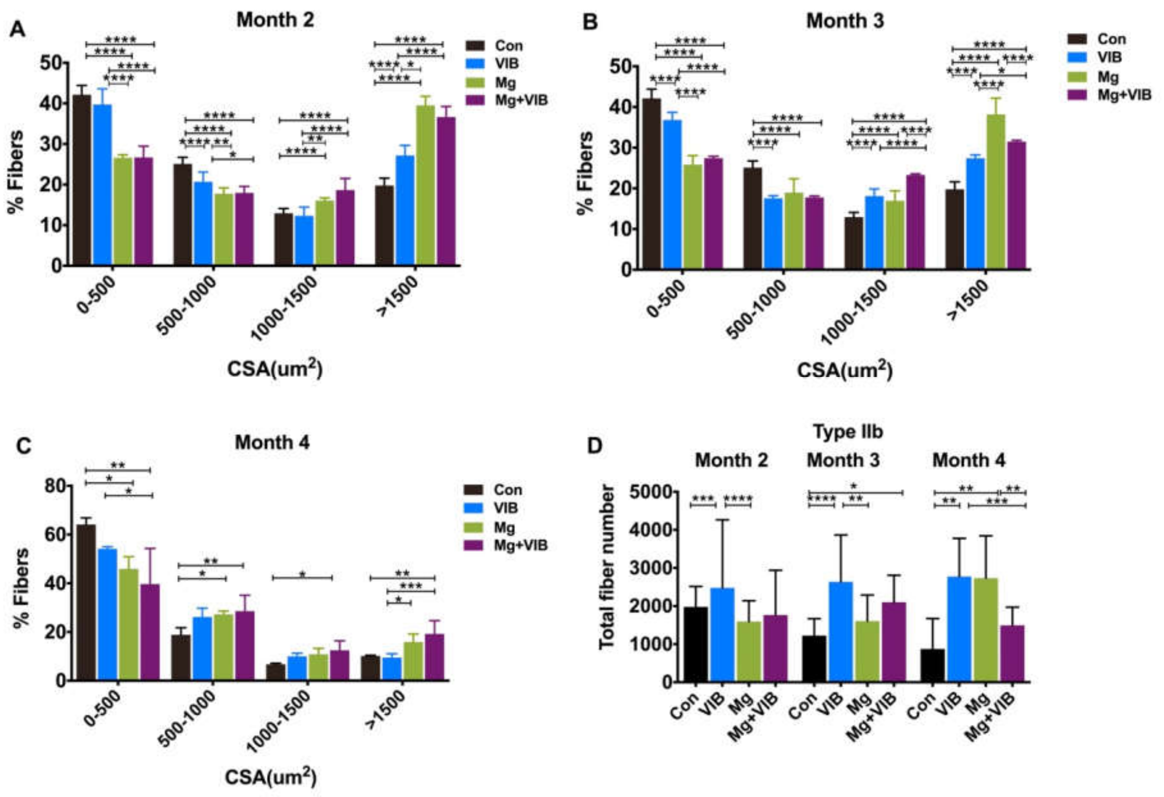

Figure S2. (A-C) Mg and Mg+VIB groups showed significantly higher fiber percentage with CSA between 1000-1500 um^2 and >1500 um^2 than Con group, while VIB and Con groups showed higher fiber percentage with CSA between 0-500 um^2 and 500-1000 um^2 at month 2. VIB, Mg and Mg+VIB groups showed significantly higher fiber percentage with CSA between 1000-1500 um^2 and >1500 um^2 than Con group at month 3. Mg+VIB group showed significantly higher fiber percentage with CSA between 1000-1500 um^2 and >1500 um^2 than Con group at month 4. (D) Type IIb fiber number in Mg group was higher than Con and Mg+VIB groups at month 4 (both at $p<0.01$), while fiber number of type IIb in VIB group was higher than Con group at month 3 and month 4 ($p<0.0001$ and $p<0.01$) *$p<0.05$, **$p<0.01$, ***$p<0.001$, and ****$p<0.0001$, n=3-5.

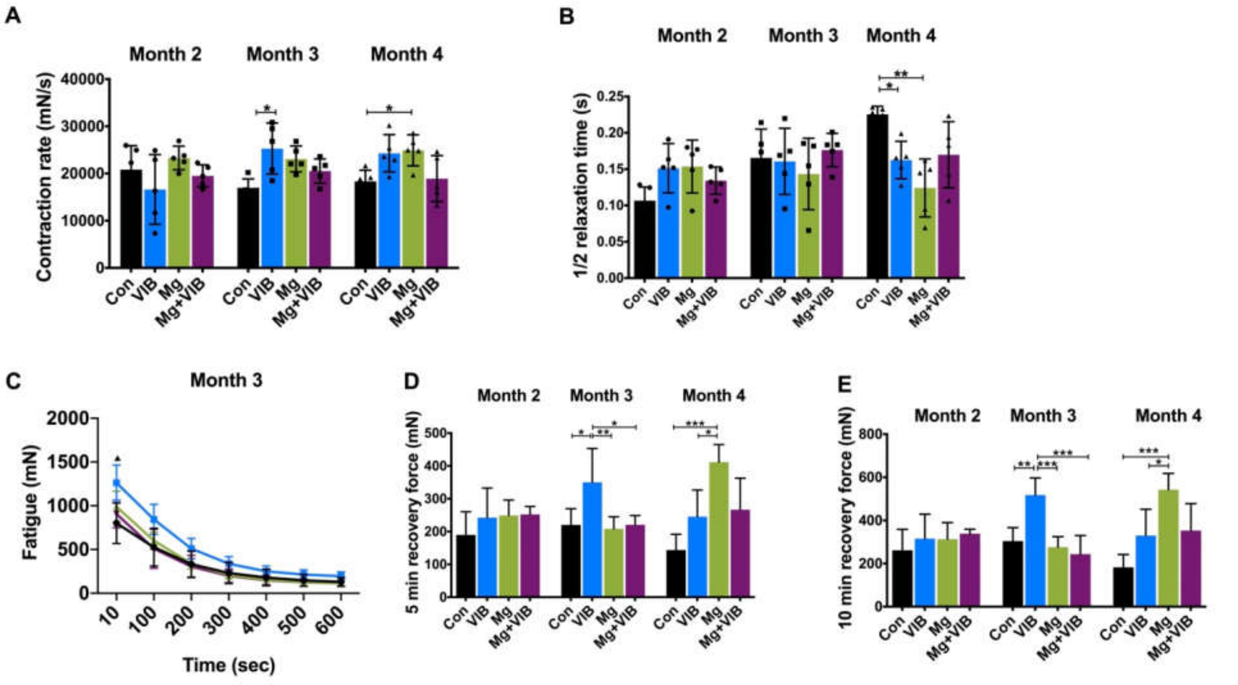

Figure S3. (A) Contraction rate of Con group was significantly lower than VIB treatment at month 3 and significantly lower than Mg group at month 4. (B) Half relation time of Mg treatment was significantly shorter than Con group at month 4. (C) VIB group showed higher fatigue curve and significantly higher force value than Con group at 10 second timepoint at month 3. ▲p<0.05 (D) 5 min recovery force and (E) 10 min recovery force of VIB group was significantly higher than Con group, Mg group and Mg+VIB group at month 3, 5 min recovery force and 10 min recovery force of Mg group was significantly higher than Con and VIB groups at month 4. *p<0.05, **p<0.01 and ***p<0.001, n=5.

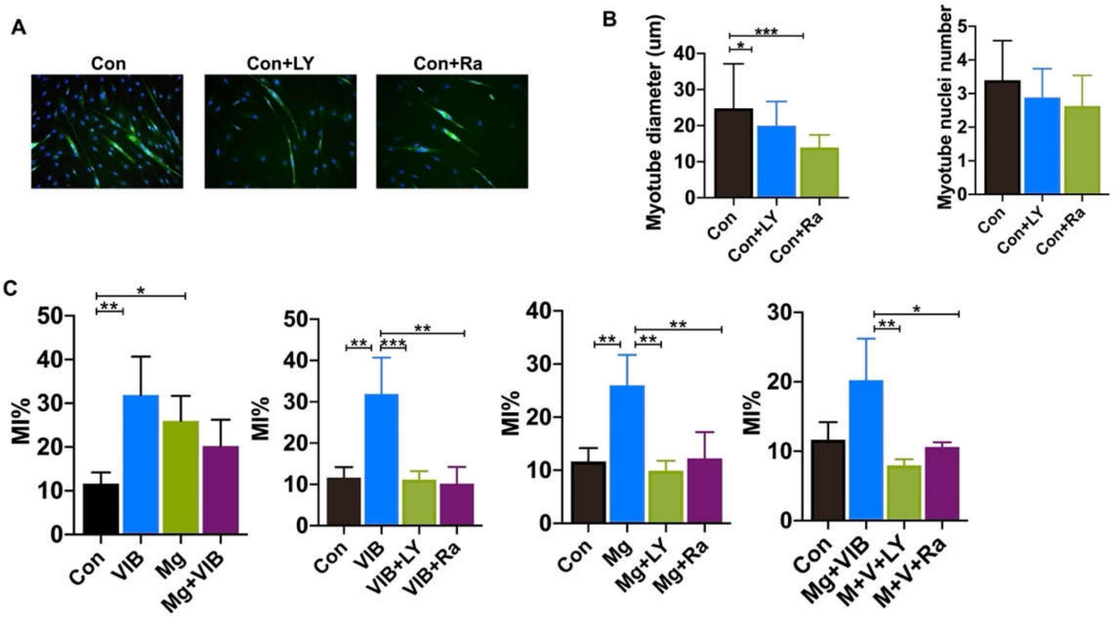

Figure S4. (A) Myoblast differentiation and myotube formation in C2C12 cell line after different treatments with PI3k/Akt inhibitor (LY294002) and mTOR inhibitor (Rapamycin). (B) Con+LY and Con+Ra groups had significantly lower myotube diameter than Con group. (C) MI of VIB and Mg groups were significantly higher than Con group. VIB+LY and VIB+Ra groups had significantly lower MI than VIB group. Mg+LY and Mg+Ra groups had significantly lower MI than Mg group. M+V+LY and M+V+Ra groups also significantly decreased MI than Mg+VIB group. *p<0.05, **p<0.01 and ***p<0.001.

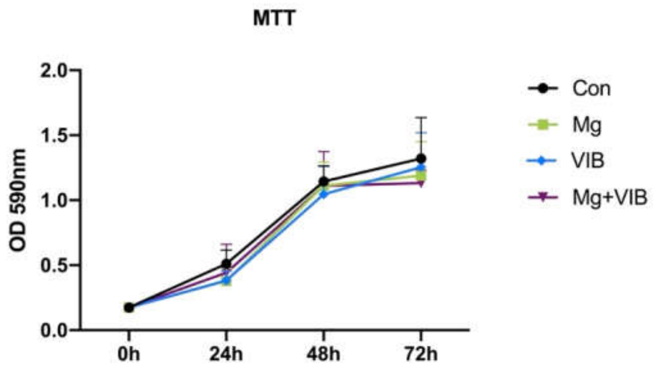

Figure S5. The C2C12 cell viability was evaluated using MTT test. The viability of C2C12 cells were increased after different treatments. VIB treatment, Mg treatment, and Mg+VIB treatment did not cause significant cell death.

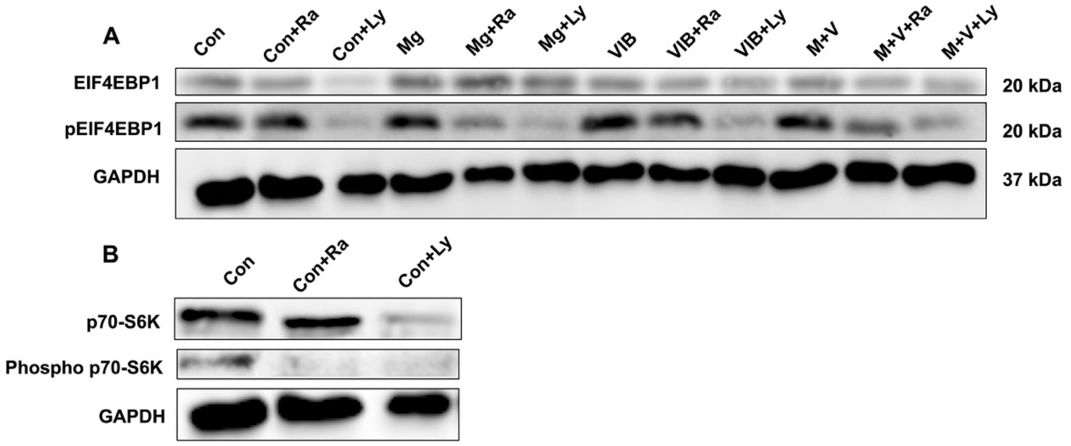

Figure S6. Western blots of protein expressions of EIF4EBP1, phospho EIF4EBP1, p70-S6K and phospho p70-S6K in C2C12. (A) Mg+LY group showed significantly lower pEIF4EBP1 than Mg group; VIB+LY group showed significantly lower pEIF4EBP1 than VIB group; M+V+LY group showed significantly lower pEIF4EBP1 than M+V group. (B) The expression level of phospho p70-S6K was significantly decreased in Con+Ra and Con+Ly groups.
